# Low shear stress protects chondrocytes from IL-1β-induced apoptosis by activating ERK5/KLF4 signaling and negatively regulating miR-143-3p

**DOI:** 10.1186/s13018-024-05140-w

**Published:** 2024-10-15

**Authors:** Jun Zhao, Yayi Xia

**Affiliations:** 1 Department of Orthopaedics, Lanzhou University Second Hospital, #82 Cuiyingmen, Lanzhou, Gansu 730000 China; 2 Orthopaedics Key Laboratory of Gansu Province, Lanzhou, Gansu China

**Keywords:** Fluid shear stress, MiR-143-3p, Chondrocyte, Apoptosis, Extracellular signal-regulated kinase 5

## Abstract

**Objective:**

This study investigated the protective effects of low fluid shear stress (FSS ≤ 2 dyn/cm²) against interleukin-1β (IL-1β)-induced chondrocyte apoptosis and explored the underlying molecular mechanisms.

**Methods:**

Chondrocytes were cultured under four conditions: control, IL-1β stimulation, low FSS, and combined low FSS + IL-1β stimulation. Apoptosis was assessed using Hoechst staining and flow cytometry. Western blotting determined the expression of caspase-3 (CASP3), caspase-8 (CASP8), and NF-κB p65. Quantitative real-time PCR measured miR-143-3p expression. The roles of miR-143-3p and the extracellular signal-regulated kinase 5 (ERK5)/Krüppel-like factor 4 (KLF4) signaling pathway were further investigated using miR-143-3p mimics and inhibitors, an ERK5 inhibitor, and a KLF4 overexpression vector.

**Results:**

IL-1β induced significant chondrocyte apoptosis, which was markedly inhibited by low FSS. Mechanistically, low FSS suppressed miR-143-3p expression, thereby enhancing ERK5 signaling. This activated ERK5 subsequently upregulated KLF4 expression, further mitigating IL-1β-induced damage. Importantly, miR-143-3p overexpression under low FSS conditions exacerbated IL-1β-induced apoptosis, while miR-143-3p inhibition attenuated it. Consistent with this, ERK5 inhibition augmented IL-1β-induced apoptosis, whereas KLF4 overexpression suppressed it.

**Conclusion:**

Low FSS protects chondrocytes from IL-1β-induced apoptosis by suppressing miR-143-3p and activating the ERK5/KLF4 signaling pathway. This study reveals a novel mechanism by which mechanical stimulation protects cartilage.

## Introduction

Osteoarthritis (OA) is a highly prevalent joint disease characterized by cartilage degradation, bone changes, and joint pain [1]. Obesity, joint injuries, and abnormal mechanical forces are significant risk factors for developing OA [2]. Left untreated, OA can impose immense economic burdens at both the individual and healthcare system levels [3]. Research has shown that compared to demographically matched non-OA controls, patients with knee OA on average incur significantly higher healthcare costs due to increased healthcare utilization, greater medication use, and more missed workdays resulting from higher disability levels [4]. With the growing number of OA patients worldwide, the economic burden on medical insurance and healthcare systems is projected to rise considerably [5].

Fluid shear stress (FSS) is an important biophysical factor that cells within diarthrodial joints experience constantly with body movement. The level and pattern of FSS has significant effects on chondrocyte metabolism and mesenchymal stem cell (MSC) behavior [6]. Prior in vitro studies applying well-defined FSS to chondrocytes and stem cells have provided critical insights into mechanotransduction pathways [7]. For chondrocytes, both excessive and too little FSS can disturb homeostasis, with aberrant levels triggering inflammatory and catabolic responses through molecular signals such as microRNAs [8]. Studies have shown that excessive FSS can lead to a more than 10-fold upregulation of H3K4me3, which in turn targets ZBTB20, ultimately activating Wnt signaling and contributing to cartilage degeneration [9]. This highlights the importance of epigenetic mechanisms in FSS-mediated cartilage damage. Interestingly, moderate FSS may protect cartilage through upregulation of transcription factors like KLF4 [6]. For mesenchymal stem cells, the rate of applied FSS influences intracellular calcium handling and cytoskeletal remodeling, determining whether the cells commit to osteogenic or chondrogenic lineages [7]. However, the interplay between FSS and biomaterials in directing MSC differentiation remains an active area of research. The use of biomaterial scaffolds, such as collagen microcarriers or nanocomposite xerogels, in conjunction with FSS, can enhance MSC viability, migration, and osteogenic differentiation [10]. Specifically, the combination of mechanical FSS with collagen microcarriers promotes MSC viability and migration, while the addition of osteogenic medium and biomaterials (B30 and B30Str) increases alkaline phosphatase activity and upregulates the expression of osteogenic markers such as Runx2 and ALP [10]. This suggests that FSS, in combination with appropriate biomaterials, can significantly influence the differentiation potential of MSCs, although further research is needed to fully elucidate the mechanisms involved in chondrogenic differentiation. Additionally, FSS-stimulated chondrocytes secrete factors promoting subchondral bone resorption [11]. While past work established roles for shear stress magnitude and rate, the exact epigenetic and molecular mechanisms involved require further elucidation. This project aims to address this knowledge gap and advance understanding of FSS-mediated joint mechanobiology using reproducible mechanical loading devices.

MiR-143-3p is a versatile microRNA (miRNA) that plays distinct roles in different diseases. It has been extensively studied in cancer where downregulation promotes tumor development by enhancing proliferation [12, 13]. For instance, in Alzheimer’s disease miR-143-3p inhibits amyloid precursor protein (APP) phosphorylation and beta-amyloid (Aβ) generation by targeting death-associated protein kinase 1 (DAPK1) [14]. However, in rheumatoid arthritis (RA) miR-143-3p expression is upregulated. Analysis of synovial tissue from RA patients shows higher levels versus OA controls [15]. In vitro, miR-143-3p activates the TNF-α mediated Ras/p38 MAPK signaling pathway by targeting insulin-like growth factor 1 receptor (IGF1R) and insulin-like growth factor binding protein 5 (IGFBP5), impacting proliferation [15]. Previous research has demonstrated a link between miR-143-3p and EIF2 signaling pathways relevant to cell survival, suggesting a potential role in the context of OA [16]. In the OA inflammatory environment, miR-143-3p may modulate stem cell survival and differentiation, participating in pathogenesis. In summary, miR-143-3p is a multifaceted miRNA with distinct expression profiles and mechanisms in OA warranting further study.

The role of ERK5 in osteoarthritis (OA) is complex and not fully understood, but emerging evidence points towards a protective role in cartilage homeostasis. While some studies suggest ERK5 suppresses chondrogenesis by downregulating Col2a1 and Sox9, key chondrocyte differentiation factors [17], other findings show that MEK5/ERK5 knockdown in mesenchymal stem cells (MSCs) actually increases SOX9 and COL2A1 expression, along with other cartilage-specific markers [18]. Conditional ERK5 loss in mesenchymal cells or chondrocytes, respectively, resulted in delayed chondrogenesis with a lack of chondrocyte hypertrophy [19] and impaired endochondral ossification leading to growth defects and bone loss [20]. These findings indicate ERK5 is not simply suppressive but crucial for the timely induction of chondrocyte hypertrophy, essential for cartilage development and maintenance. Therefore, despite some conflicting data, the overall evidence suggests ERK5 plays a protective role, with its proper function being essential for healthy cartilage. Further research is needed to clarify ERK5’s intricate interactions with other pathways, such as CDC42 and those targeted by miR143-3p, in OA pathogenesis.

This study aims to investigate the protective effect and underlying molecular mechanisms of low FSS (≤ 2 dyn/cm2) on IL-1β-induced chondrocyte apoptosis. We hypothesize that low FSS can suppress chondrocyte apoptosis induced by IL-1β through the regulation of specific microRNAs and their target signaling pathways. Mechanistically, we identified extracellular signal-regulated kinase 5 (ERK5) as a target gene of hsa-miR-143-3p using dual-luciferase reporter assays. Downregulation of miR-143-3p under low FSS conditions led to enhanced activation of the ERK5/KLF4 signaling pathway, which further inhibited the IL-1β-induced damage to chondrocytes. Understanding the role of low FSS in modulating chondrocyte response to inflammatory stimuli may provide new insights into the mechanobiology of cartilage and potential therapeutic strategies for osteoarthritis.

## Materials and methods

### Cell culture and low FSS loading

Human SW1353 cells obtained from Fuheng Biology were cultured in DMEM containing 10% FBS and 1% penicillin/streptomycin at 37 °C with 5% CO2. Low FSS (1.8 dyn/cm2) was applied based on Zhou‘s research [21], using a rocking system with 8-well dishes (37.6 × 27.9 mm, 1.86mL medium) rocking 10° at 60 cycles/minute. FSS was quantified in this system. Cells were serum-starved in DMEM for 8 h prior to FSS loading. The duration for FSS loading was 1 h. The concentration of cells incubated with IL-1b was 10 ng/ml for 4 h. If there was a group that needs to add IL-1 β to induce apoptosis, it was necessary to add IL-1 β for 4 h, and then load the fluid shear force.

### Cell transfection

SW1353 cells were transfected following the manufacturer’s protocol. Cells were transfected with miR-143-3p mimic, mimic negative control (NC) (RiboBio, China), inhibitor or inhibitor NC (RiboBio, China) using Lipofectamine 2000 (Invitrogen, USA). After 48 h, cells were transfected with ERK5 overexpression vector pcDNA3.1-ERK5A (GenePharma, China), ERK5 siRNA (GenePharma, China), KLF4 overexpression vector pcDNA3.1-KLF4A (GenePharma, China) or KLF4 siRNA (GenePharma, China). The concentrations of siRNA and plasmid used were 100 nM and 200 ng/µl, respectively. Transfected cells were collected for subsequent experiments.

### Detection of apoptosis with hoechst staining

Each group of cells was stained using a Hoechst33258 (Beyotime, China) kit following the manufacturer’s instructions: to perform Hoechst staining to detect apoptotic cells, first clean the coverslips with 70% ethanol for at least 5 min. Wash them 3 times with sterile PBS or 0.9% NaCl, and once with cell culture medium. Place the coverslips in 6-well plates and seed cells to reach 50–80% confluency overnight. After inducing apoptosis, remove the medium and fix the cells with 0.5 ml of fixative solution for at least 10 min (or overnight at 4 °C). Wash twice with PBS or NaCl, 3 min each time, remove the liquid and shake gently during the washes. Add 0.5 ml of Hoechst 33,258 dye and stain for 5 min with gentle shaking. Wash twice again with PBS or NaCl to remove excess dye. Mount the stained coverslip onto a glass slide using antifade mounting medium, avoiding bubbles. Cells were observed under a fluorescence microscope with excitation at 350 nm and emission at 460 nm to detect apoptotic nuclei stained blue by Hoechst 33,258.

### Apoptosis analysis by flow cytometry

In this study, we used the Annexin V/7-AAD staining method combined with flow cytometry to detect cell apoptosis. Since the transfected plasmids have fluorescent markers, we chose 7-AAD instead of PI to avoid interference with the FITC channel. This method utilizes the specific binding of Annexin V to the phosphatidylserine (PS) exposed on the surface of apoptotic cells, as well as the staining characteristics of 7-AAD for the cell nucleus, to distinguish apoptotic cells, necrotic cells, and normal cells. To detect apoptotic cells, cells were collected by centrifugation and washed with incubation buffer. The cells were then resuspended in labeling solution containing Annexin V-FITC and incubated at room temperature for 10–15 min. After centrifugation and washing, 7-AAD solution was added, and the cells were incubated for 20 min at 4 °C in the dark with periodic shaking. Analysis was performed on a flow cytometer using 488 nm excitation and detection filters for FITC (515 nm) and 7-AAD (647 nm), allowing us to discriminate apoptotic from normal cells.

### Quantitative real-time PCR (qRT-PCR)

Total RNA was isolated from SW1353 cells using Trizol reagent (Thermo Fisher, America). The concentration and purity of total RNA were determined with a spectrophotometer. RNA samples were reverse transcribed to cDNA using a synthesis kit (Accurate Biology, China) according to the manufacturer’s instructions. Glyceraldehyde-3-phosphate dehydrogenase (GAPDH) was used as the reference gene for mRNA analysis. Expression levels were detected using a CFX96 real-time fluorescence quantitative PCR detection system (BIO-RAD, America) and SYBR Green kit (Accurate Biology, China). The 2^−ΔΔCt^ method was used to quantify mRNA and miRNA expression. The miRNA primer sequence was from the miRNA cDNA synthesis kit (Accurate Biology, China) and miRNA RT-qPCR SYBR kit (Accurate Biology, China). Target gene primers are listed in Table 1.

**Table 1:** Target gene primers used for qRT-PCR

Name	Sequence (5ʹ-3ʹ)
GAPDH-F	GGAGCGAGATCCCTCCAAAAT
GAPDH-R	GGCTGTTGTCATACTTCTCATGG
U6-F	GAGAAGATTAGCATGGCCC
U6-R	AATATGGAACGCTTCACGA
miR-143-3p RT	GTCGTATCCAGTGCAGGGTCCGAGGTATTCGCACTGGATACGACGAGCTA
miR-143-3p-F	CGCGTGAGATGAAGCACTG
miR-143-3p-R	AGTGCAGGGTCCGAGGTATT
CASP3-F	CATGGAAGCGAATCAATGGACT
CASP3-R	CTGTACCAGACCGAGATGTCA
CASP 8-F	TTTCTGCCTACAGGGTCATGC
CASP8 -R	GCTGCTTCTCTCTTTGCTGAA
MMP13-F	TCCTGATGTGGGTGAATACAATG
MMP13-R	GCCATCGTGAAGTCTGGTAAAAT
RELA(NF-κB p65)-F	GTGGGGACTACGACCTGAATG
RELA(NF-κB p65)-R	GGGGCACGATTGTCAAAGATG

### Luciferase assay

The potential binding sites of ERK5 to miR143-3p were predicted using TargetScan Human 7.2 (https://www.targetscan.org/). The wild type (WT) or mutant (MUT) 3’UTR of ERK5 fused to a dual luciferase reporter vector (GenePharma, China) was co-transfected with hsa-miR143-3p mimic, inhibitor or negative control (RiboBio, China) into 293T cells using Lipofectamine 2000 (Invitrogen, USA). After 48 h, cells were harvested and the luciferase activity was measured using a luciferase detection system (Promega, USA).

### Western blot analysis

Total protein from SW1353 cells was extracted according to the protein extraction kit (Thermo Fisher, USA) instructions. The protein concentration was measured using a BCA kit (Solarbio, China). Target proteins were separated by sodium dodecyl sulfate polyacrylamide gel electrophoresis (SDS-PAGE) and transferred to a polyvinylidene fluoride (PVDF) membrane. The membrane was blocked with Tris-buffered saline containing 5% skim milk for 2 h at room temperature. It was then incubated overnight at 4 °C with primary antibodies against CASP3 (1:1000, Abcam, USA), CASP8 (1:1000, Abcam, USA), NF-κB p65 (1:1000, Abcam, USA) and GAPDH (1:1000, Abcam, USA). Next, a horseradish peroxidase-conjugated secondary antibody (1:2000; Abcam, USA) was applied for 1 h at room temperature. Protein bands were detected using a VersaDoc imaging system (Bio-Rad, USA). In the experiment, it was found that the imaging of some proteins such as CASP8 was difficult, while the band gray value of marker is high, which affected the gene imaging. So we cut the relevant marker strips to promote the imaging of certain proteins, which in itself did not affect the specific experimental results. In order to save experimental time and materials, some bands were washed with membrane washing buffer, re-incubated with new antibodies, and then re-luminescent and exposed with ECL. If the same membrane needed to detect another protein at the same time, we needed to wash the membrane with membrane washing solution for 30 min, then sealed it with 5% skim milk, and re-incubateed to test the first and second antibodies of another protein. As the long experimental time and the change of experimental site, most of the protein imprinted bands were photographed by automatic WB imager, and some are exposed by chemiluminescence and X-ray film. The specific operation of the latter is as follows: take out the rinsed NC film, transfer the film to the exposure cassette, make it fully contacted by ECL working liquid (completely covered), X-ray film on the wrapped NC film, press, develop, fix and develop. The exposed film is first developed in the developer until the strip appears, usually within the 1 min, and the background will be deepened if the time is too long. Rinse in the fixing solution for ten seconds, and the protein band can be clearly displayed on the X-ray film.

### The hypothesis and the corresponding test

Hypotheses : 1.IL-1β induced apoptosis in chondrocytes. 2. Low fluid shear stress (FSS) inhibited IL-1β-induced apoptosis in chondrocytes. 3. FSS suppressed the secretion of miR-143-3p in chondrocytes and then inhibited IL-1β-induced apoptosis of chondrocytes. 4. MiR-143-3p attenuated the protective effects of the ERK5 signaling pathway against IL-1β-induced apoptosis in chondrocytes.5. KLF4, downstream of the ERK5 signaling pathway, suppressed IL-1β-induced apoptosis in chondrocytes. 6. A potential target of hsa-miR-143-3p was ERK5. The independent sample T test was used for the comparison between the two groups of hypothesis1-5, and the one-way ANOVA was used for the comparison of three groups and more than three groups. Hypothesis 6 used paired sample T-test. Independent sample T-test needs to meet the following conditions: (1) Independence, that is, the observations are independent of each other. (2) Normality, each sample comes from the population of normal distribution. One-way ANOVA data need to meet three basic conditions: (1) Independence: the object of observation is an independent random sampling from all levels of the factors studied. (2) Normality: the dependent variables at each level should obey the normal distribution. (3) Homogeneity of variance: the population at all levels has the same variance. Paired sample T-test needs to meet the following requirements: (1) The dependent variable accords with normal distribution; (2) Observation independence: each observation is independent of each other; (3) Dependent variables are measured at an incremental level, such as a ratio or interval. (4) The argument must consist of two related groups or matching pairs.

### Statistical analysis

Data are presented as mean ± standard deviation. GraphPad Prism 9 (GraphPad Software Inc., USA) was used for data analysis and visualization. Statistical analysis was performed using unpaired t-tests or one-way ANOVA, as appropriate. *P* < 0.05 was considered statistically significant.

## Results

### IL-1β was found to induce apoptosis in chondrocytes

**Fig. 1:**
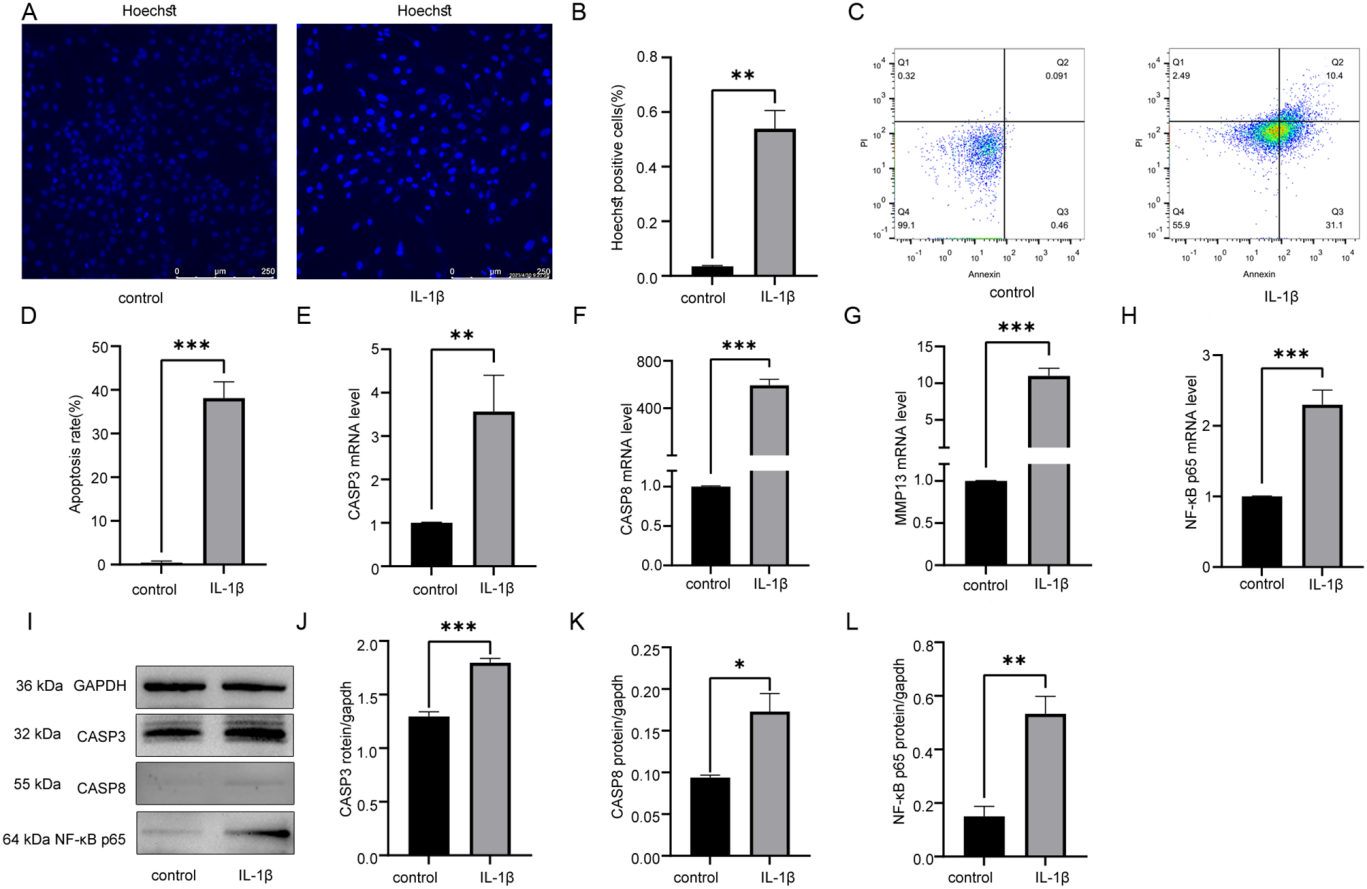
IL-1β induced apoptosis in chondrocytes. (**A**) Comparison of apoptotic cells by Hoechst staining (Hoechst33258) between IL-1β and control groups. (**B**) Statistical results of apoptotic cell proportion in (**A**). (**C**) Comparison of apoptotic cells by flow cytometry between IL-1β and control groups. (**D**) Statistical results of apoptotic cell proportion in (**C**). (**E**) Statistical results of relative CASP3 mRNA by qRT-PCR between groups. (**F**) Statistical results of relative CASP8 mRNA by qRT-PCR between groups. (**G**) Statistical results of relative MMP13 mRNA by qRT-PCR between groups. (**H**) Statistical results of relative NF-κB p65 mRNA by qRT-PCR between groups. (**I**-**L**) Western blot analysis and statistical results of CASP3, CASP8 and NF-κB p65 protein levels. Statistical results presented as mean ± SD of three independent experiments. ns: No significance. *p*<0.05*, *p*<0.01**, *p*<0.001***.MMP13, Matrix metallopeptidase 13

To study the effects of IL-1β on chondrocyte apoptosis, Hoechst staining (Fig. 1A-B), flow cytometry (Fig. 1C-D), qRT-PCR (Fig. 1E-H), and Western blot analysis (Fig. 1I-L) were performed between IL-1β-treated and control groups. The results demonstrated that IL-1β could induce chondrocyte apoptosis.

### Low fluid shear stress (FSS) was found to attenuate IL-1β-induced apoptosis in chondrocytes

**Fig. 2:**
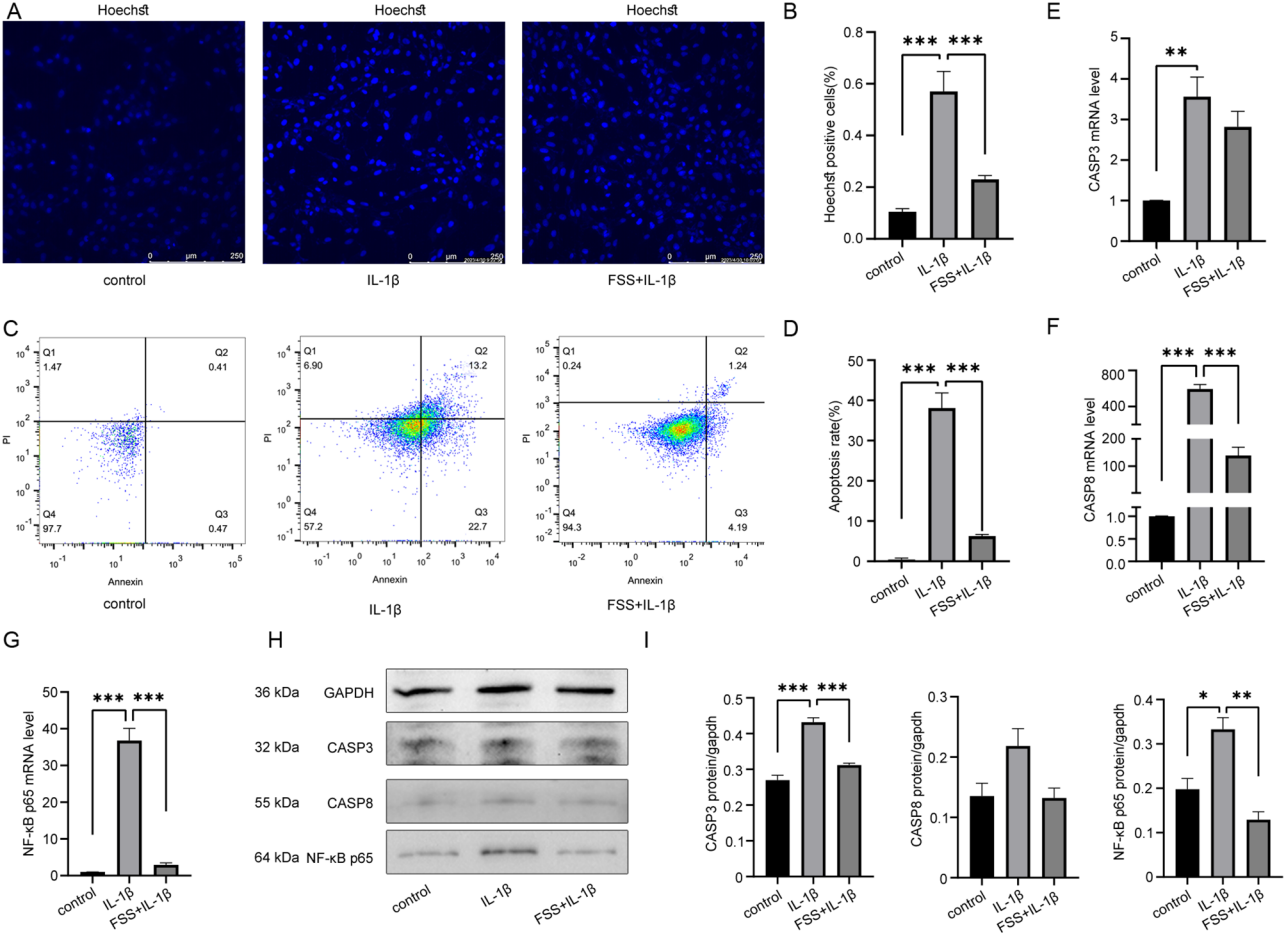
Low FSS suppressed IL-1β-induced apoptosis in chondrocytes. (**A**) Comparison of apoptotic cells among control, IL-1β and low FSS + IL-1β groups by Hoechst staining (Hoechst33258). (**B**) Statistical results of apoptotic cell proportion in (**A**). (**C**) Comparison of apoptotic cells among groups by flow cytometry. (**D**) Statistical results of apoptotic cell proportion in (**C**). (**E**) Statistical results of relative CASP3 mRNA by qRT-PCR among groups. (**F**) Statistical results of relative CASP8 mRNA by qRT-PCR among groups. (**G**) Statistical results of relative NF-κB p65 mRNA by qRT-PCR among groups. (**H**-**I**) Western blot analysis and statistical results of CASP3, CASP8 and NF-κB p65 protein levels. Statistical results presented as mean ± SD of three independent experiments. ns: No significance. *p*<0.05*, *p*<0.01**, *p*<0.001***

To investigate the effects of low FSS on IL-1β-induced chondrocyte apoptosis, Hoechst staining (Fig. 2A-B), flow cytometry (Fig. 2C-D), qRT-PCR (Fig. 2E-G), and Western blot analysis (Fig. 2H-I) were performed among IL-1β, low FSS + IL-1β, and control groups. Statistical analysis revealed that low FSS decreased IL-1β-induced chondrocyte apoptosis.

### Low fluid shear stress (FSS) was found to enhance the activation of the ERK5/KLF4 signaling pathway, and downregulate the expression of miR-143-3p

**Fig. 3:**
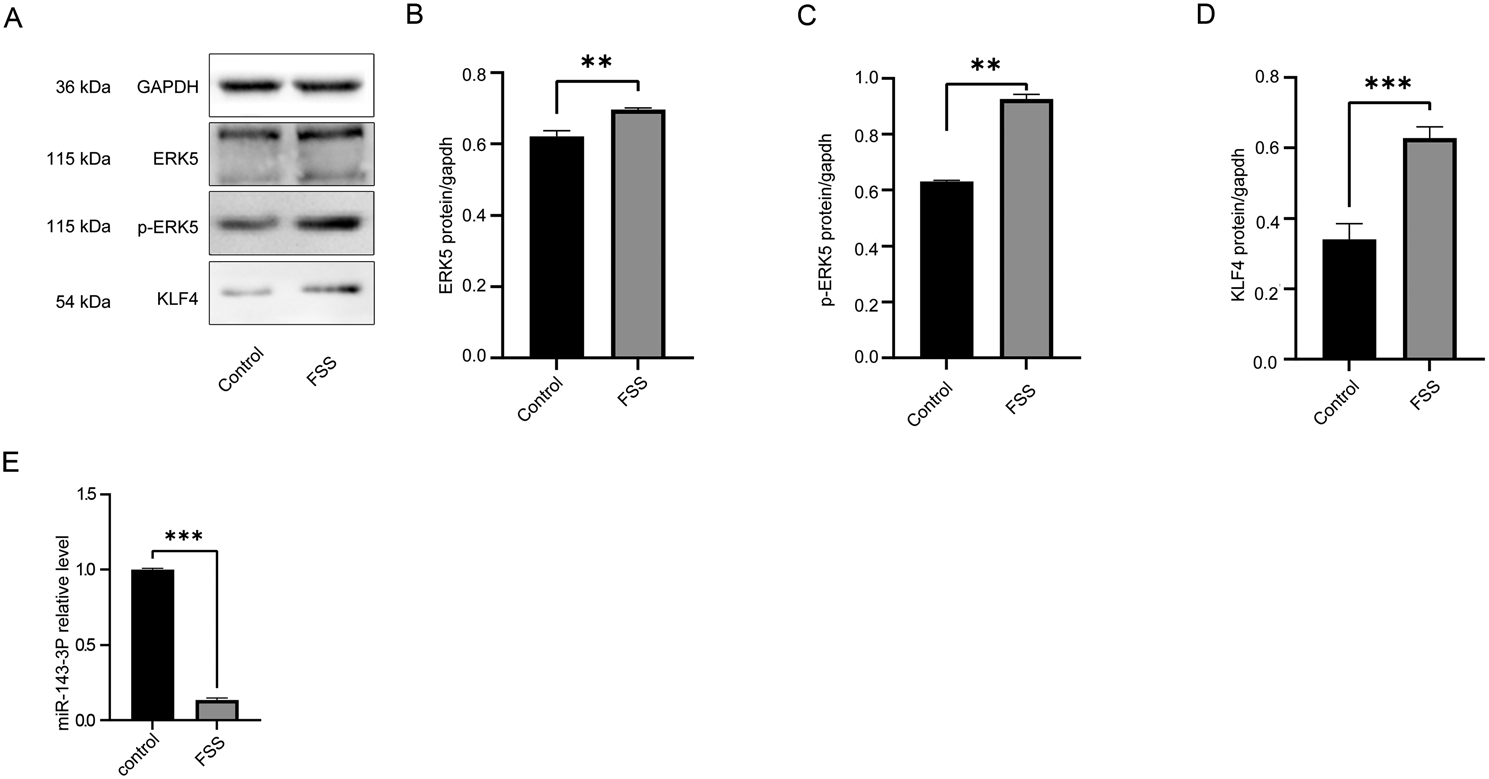
Low FSS enhanced the activation of the ERK5/KLF4 signaling pathway, and downregulated the expression of miR-143-3p. (**A**-**D**) Western blot analysis and statistical results of ERK5, phosphorylated ERK5, and KLF4 between the low FSS group and control group. (**E**) Statistical results of relative miR-143-3p by qRT-PCR between the low FSS group and control group. Statistical results presented as mean ± SD of three independent experiments. *p*<0.05*, *p*<0.01**, *p*<0.001***

To investigate the effects of low fluid shear stress (FSS) on the ERK5/KLF4 signaling pathway and miR-143-3p expression, we employed Western blot analysis to compare the protein levels of ERK5, phosphorylated ERK5, and KLF4 between the low FSS group and control group (Fig. 3A). Additionally, we used real-time quantitative PCR to detect the expression of miR-143-3p in the low FSS group and control group. The results demonstrated that low FSS enhanced the activation of the ERK5/KLF4 signaling pathway (Fig. 3B-D) and downregulated the expression of miR-143-3p(Fig. 3E).

### MiR-143-3p exacerbated IL-1β-Induced Chondrocyte apoptosis under low fluid shear stress

**Fig. 4:**
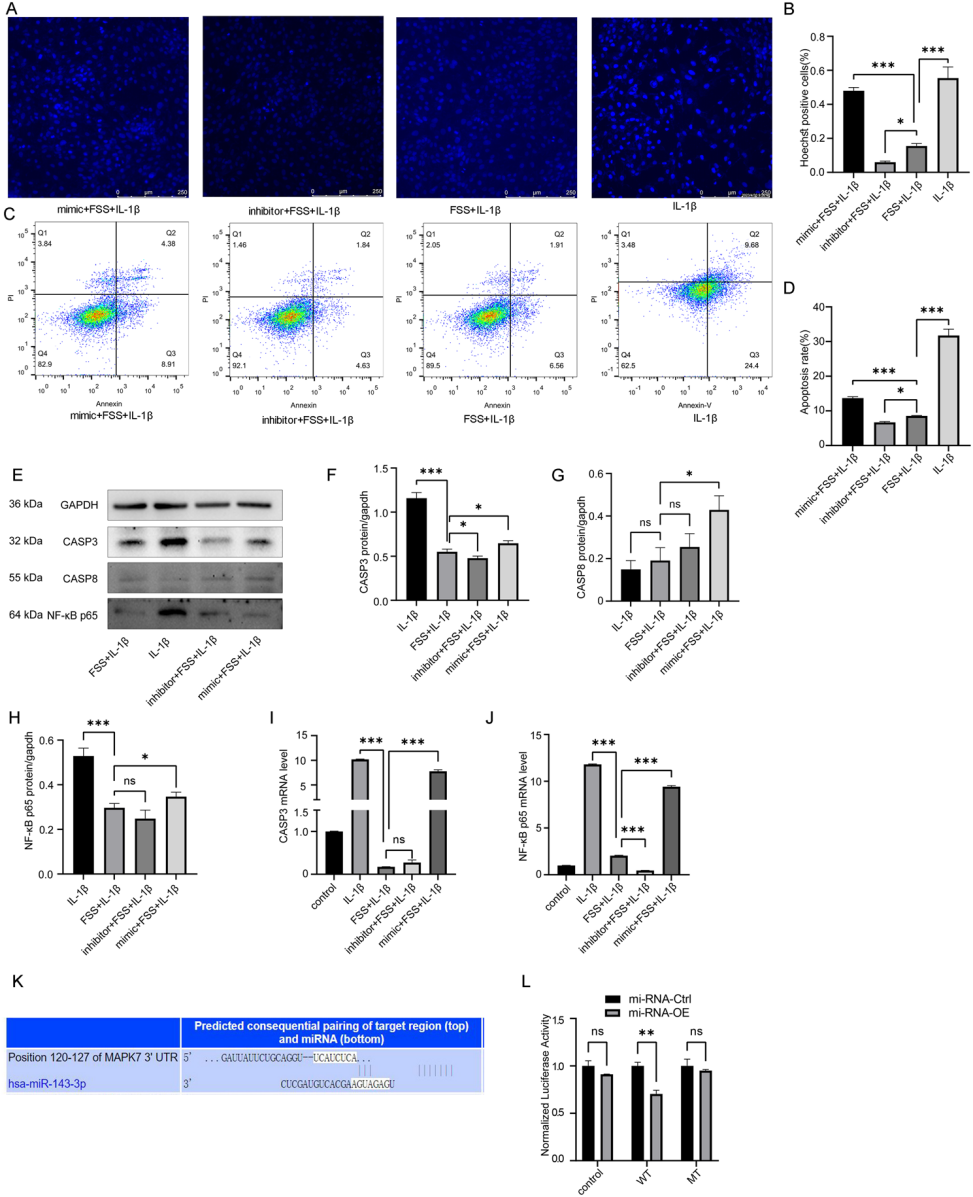
Under FSS condition, miR-143-3p exacerbated IL-1β-induced chondrocyte apoptosis. Dual-luciferase reporter confirmed miR-143-3p targeted and suppressed the expression of ERK5. (**A**) Comparison of apoptotic cells among miR-143-3p mimic + FSS + IL-1β, miR-143-3p inhibitor + FSS + IL-1β, FSS + IL-1β and IL-1β groups by Hoechst staining (Hoechst33258). (**B**) Statistical results of apoptotic cell proportion in A. (**C**) Comparison of apoptotic cells among miR-143-3p mimic + FSS + IL-1β, miR-143-3p inhibitor + FSS + IL-1β, FSS + IL-1β and IL-1β groups by flow cytometry. (**D**) Statistical results of apoptotic cell proportion in C. (**E**-**H**) Western blot analysis and statistical results of CASP3, CASP8 and NF-κB p65 protein levels. (**I**) Statistical results of relative CASP3 mRNA by qRT-PCR among groups. (**J**) Statistical results of relative NF-κB p65 mRNA by qRT-PCR among groups. (**K**-**L**)Target relationship of miR-143-3p and ERK5 by Dual-Luciferase Reporter Assay. Statistical results presented as mean ± SD of three independent experiments. WT: Wild Type. MT: Mutant. OE: Overexpression. ns: No significance. *p*<0.05*, *p*<0.01**, *p*<0.001***

Low fluid shear stress (FSS) exacerbated IL-1β-induced chondrocyte apoptosis. To determine the role of miR-143-3p in this process, we assessed apoptosis in human chondrocytes treated with IL-1β under low FSS conditions using Hoechst staining (Fig. 4 A-B) and flow cytometry (Fig. 4 C-D). These analyses revealed increased apoptosis in the miR-143-3p mimic group and decreased apoptosis in the miR-143-3p inhibitor group compared to controls (Fig. 4 A-D). Western blot analysis (Fig. 4 E-H) showed corresponding changes in the expression levels of cleaved caspase-3 and PARP, key indicators of apoptosis. Furthermore, QRT-PCR analysis (Fig. 4 I-J) demonstrated that the miR-143-3p mimic upregulated, while the inhibitor downregulated, the mRNA levels of CASP3 and NF-κB p65. Finally, a dual-luciferase reporter assay confirmed that miR-143-3p directly targeted and suppressed ERK5 expression (Fig. 4 K-L). These findings indicated that miR-143-3p exacerbated IL-1β-induced chondrocyte apoptosis under low FSS conditions, at least in part, by targeting ERK5.

### ERK5 inhibits IL-1β-Induced Chondrocyte apoptosis and mediates the Protective Effect of Fluid Shear stress

**Fig. 5:**
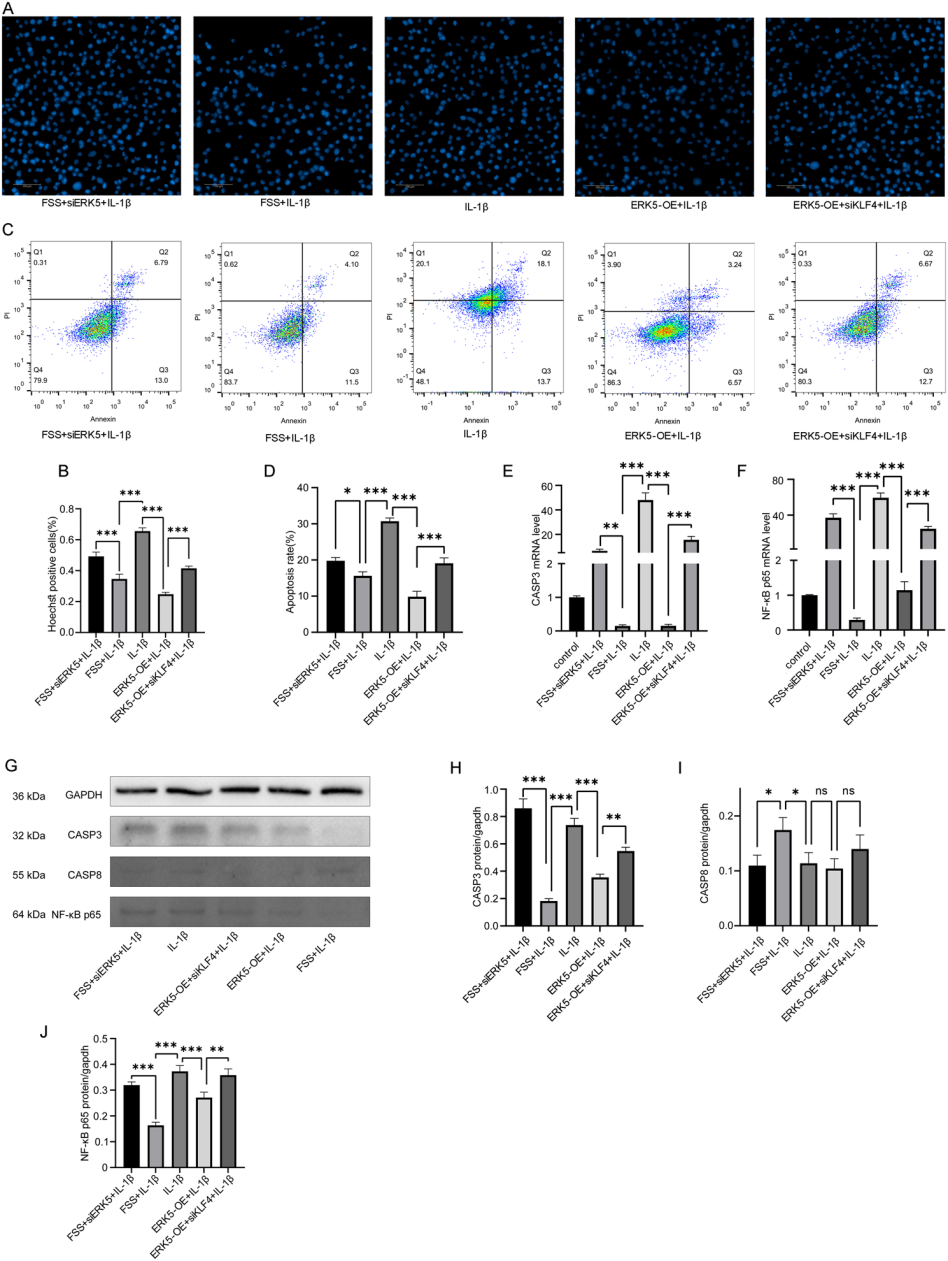
Overexpression of ERK5 inhibits IL-1β-induced chondrocyte apoptosis. The anti-apoptotic effect of low fluid shear stress on IL-1β-treated chondrocytes is reversed by ERK5 inhibitors. Inhibiting the downstream target KLF4 counteracts the protective effect of ERK5 overexpression against IL-1β-induced chondrocyte apoptosis. (**A**) Comparison of apoptotic cells among FSS + siERK5 + IL-1β, FSS + IL-1β, IL-1β, ERK5-OE + IL-1β and ERK5-OE + siKLF4 + IL-1β groups by Hoechst staining (Hoechst33258). (**B**) Statistical results of apoptotic cell proportion in A. (**C**) Comparison of apoptotic cells among FSS + siERK5 + IL-1β, FSS + IL-1β, IL-1β, ERK5-OE + IL-1β and ERK5-OE + siKLF4 + IL-1β groups by flow cytometry. (**D**)Statistical results of apoptotic cell proportion in C. (**E**) Statistical results of relative CASP-3 mRNA by qRT-PCR among groups. (**F**) Statistical results of relative NF-κB p65 mRNA by qRT-PCR among groups. (**G**-**J**) Western blot analysis and statistical results of CASP3, CASP8 and NF-κB p65 protein levels. Statistical results presented as mean ± SD of three independent experiments. ns: No significance. *p*<0.05*, *p*<0.01**, *p*<0.001***. ERK5, Extracellular signal-regulated kinase 5, OE, Overexpression

Overexpression of ERK5 inhibited IL-1β-induced chondrocyte apoptosis; however, this protective effect was reversed by ERK5 inhibitors under low fluid shear stress (FSS) conditions. Furthermore, the protective effect of ERK5 overexpression against IL-1β-induced apoptosis was reversed by simultaneous KLF4 inhibition, indicating that KLF4 acts downstream of ERK5. To further investigate the role of ERK5, we assessed apoptosis in human chondrocytes using Hoechst staining (Fig. 5 A-B) and flow cytometry (Fig. 5 C-D) in the following groups: FSS + siERK5 + IL-1β, FSS + IL-1β, IL-1β, ERK5-OE + IL-1β, and ERK5-OE + siKLF4 + IL-1β. Western blot analysis (Fig. 5 G-J) and qRT-PCR (Fig. 5 E-F) were performed on the same groups. These analyses demonstrated that ERK5 overexpression significantly inhibited IL-1β-induced chondrocyte apoptosis. Conversely, ERK5 knockdown using siRNA (siERK5) reversed the protective effect of FSS against IL-1β-induced apoptosis. Finally, inhibition of KLF4 abrogated the anti-apoptotic effect of ERK5 overexpression. These results confirm that ERK5 plays a protective role against IL-1β-induced chondrocyte apoptosis and that this protection is, at least in part, mediated through KLF4.

### Overexpression of KLF4 inhibited chondrocyte apoptosis induced by IL-1 β, and the inhibitory effect of low fluid shear stress on chondrocyte apoptosis induced by IL-1 β can be reversed by KLF4 inhibitors

**Fig. 6:**
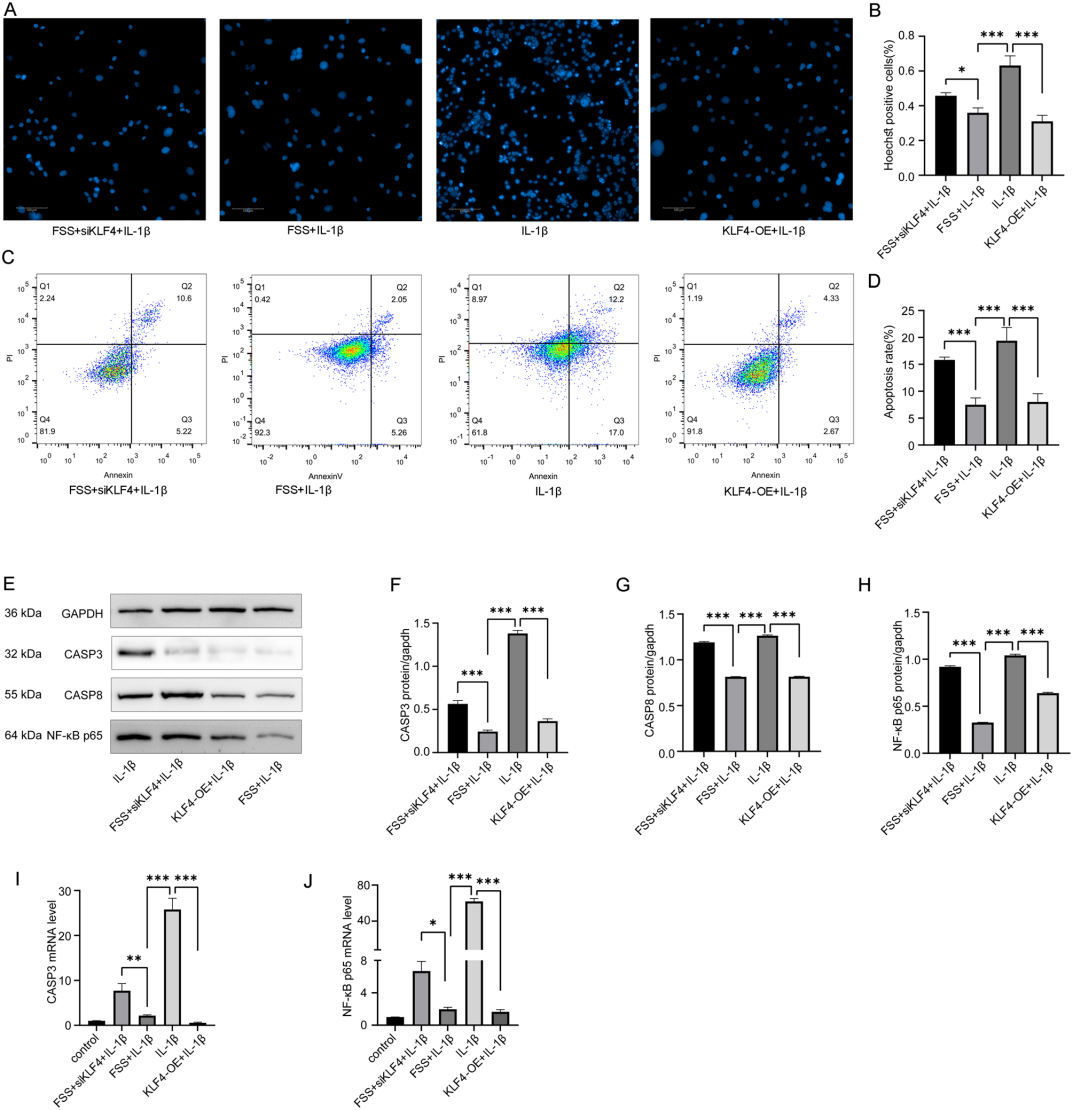
KLF4 overexpression inhibits IL-1β-induced chondrocyte apoptosis, and this anti-apoptotic effect of low fluid shear stress is reversed by KLF4 inhibitor. (**A**) Comparison of apoptotic cells among FSS + siKLF4 + IL-1β, FSS + IL-1β, IL-1β and KLF4 OE + IL-1β groups by Hoechst staining (Hoechst33258). (**B**) Statistical results of apoptotic cell proportion in A. (**C**) Comparison of apoptotic cells among FSS + siKLF4 + IL-1β, FSS + IL-1β, IL-1β and KLF4 OE + IL-1β groups by flow cytometry. (**D**) Statistical results of apoptotic cell proportion in C. (**E**-**H**) Western blot analysis and statistical results of CASP3, CASP8 and NF-κB p65 protein levels. (**I**) Statistical results of relative CASP3 mRNA by qRT-PCR among groups. (**J**) Statistical results of relative NF-κB p65 mRNA by qRT-PCR among groups. Statistical results presented as mean ± SD of three independent experiments. ns: No significance. *p*<0.05*, *p*<0.01**, *p*<0.001***. KLF4, Krüppel-like factor 4, OE, Overexpression

To examine the role of KLF4 in IL-1β-induced chondrocyte apoptosis under FSS condition, Hoechst staining (Fig. 6A-B) and flow cytometry (Fig. 6C-D) were performed among FSS + siKLF4 + IL-1β, FSS + IL-1β, IL-1β and KLF4 OE + IL-1β groups. Western blot analysis (Fig. 6E-H) and qRT-PCR (Fig. 6I-J) were performed among FSS + siKLF4 + IL-1β, FSS + IL-1β, IL-1β and KLF4 OE + IL-1β groups. Statistical analysis demonstrated that KLF4 overexpression inhibits IL-1β-induced chondrocyte apoptosis, and this anti-apoptotic effect of low fluid shear stress is reversed by KLF4 inhibitor.

## Discussion

**Fig. 7:**
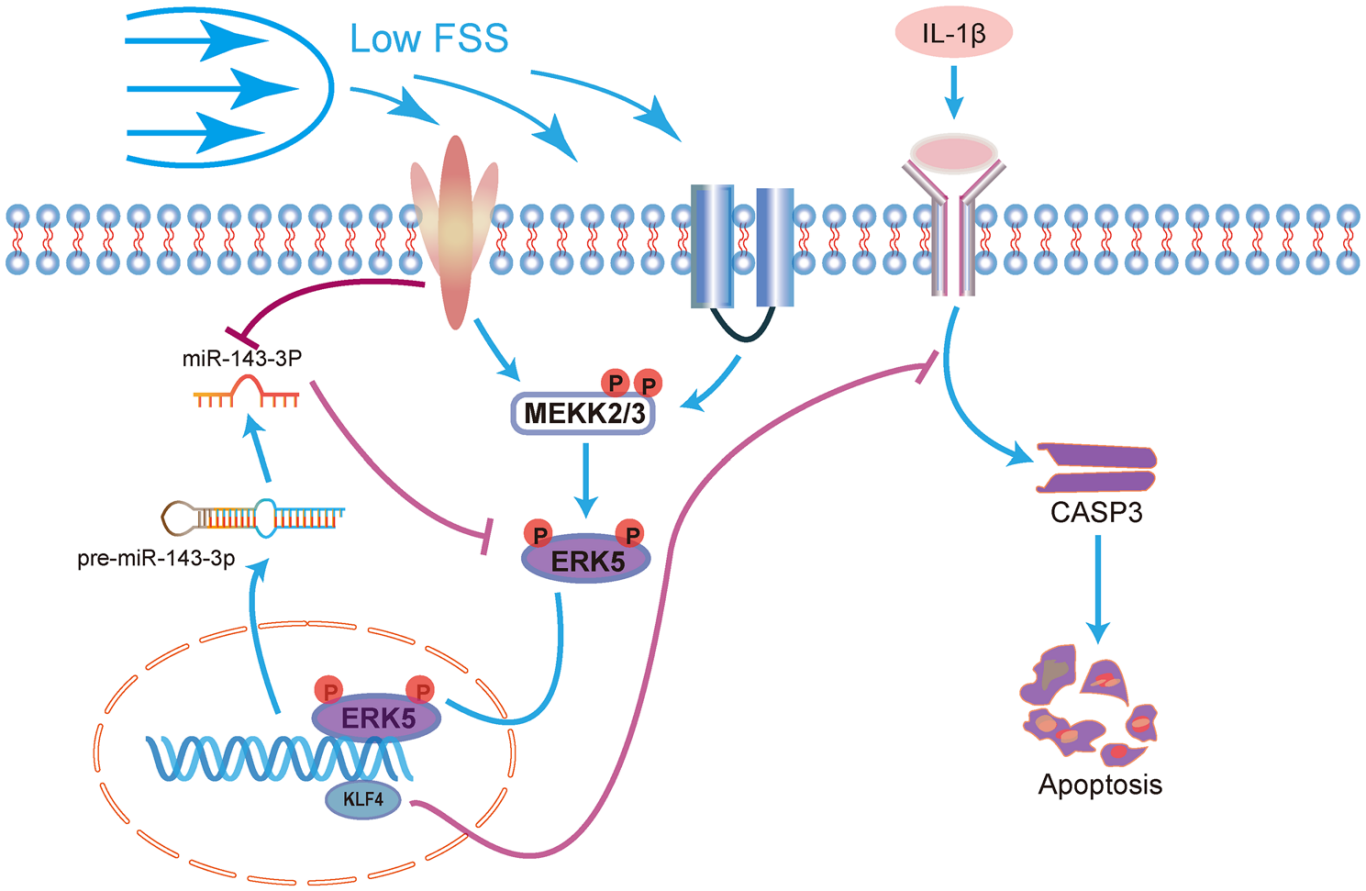
The possible mechanism of low fluid shear stress inhibiting IL-1 β-induced apoptosis of chondrocytes through miR-143-3P and ERK5/KLF4 signaling pathway

Interleukin-1 beta (IL-1β) can induce chondrocyte apoptosis [22, 23]. However, the effects of different magnitudes of FSS on IL-1β-induced chondrocyte apoptosis remained incompletely understood, with the underlying mechanisms requiring further investigation [6]. This study demonstrated that microRNA-143-3p plays a role in suppressing IL-1β-induced chondrocyte apoptosis under low FSS. Specifically, exposure to low FSS downregulated miR-143-3p levels, correspondingly inhibiting chondrocyte apoptosis. Additionally, we showed that miR-143-3p influences chondrocyte apoptosis by targeting extracellular signal-regulated kinase 5 (ERK5). Our research indicated that downregulation of miR-143-3p via activation of the ERK5/KLF4 signaling pathway suppresses IL-1β-induced chondrocyte apoptosis. To our knowledge, this is the first study to prove that miR-143-3p plays a key role in chondrocyte apoptosis induced by IL-1 β, with ERK5 and KLF4 serving as key downstream targets in this process. A diagram of the pathways under the investigation was shown in Fig. 7.

The magnitude of FSS had diverse impacts on cells involved in cartilage and bone formation [8, 24]. Precisely controlling FSS was important for tissue engineering approaches. In chondrocytes, low FSS of 2–5 dyn/cm^2^ upregulated aggrecan and collagen gene expression [6]. This shear activated integrin and PKC signaling to promote matrix synthesis. However, high FSS exceeding 15 dyn/cm^2^ induced cell death in chondrocytes through different mechanisms. FSS of 15 dyn/cm^2^ activated MAPKs like p38 and JNK, leading to apoptosis. Even higher shear of 20 dyn/cm^2^ stimulated COX-2 and PGE2 production, which suppressed antioxidant enzymes like Nrf2 [8]. The resulting oxidative stress induced chondrocyte apoptosis [8]. Mesenchymal stem cells (MSCs) demonstrated optimal chondrogenesis at 10 dyn/cm^2^ FSS through moderate Ca^2+^ influx. Interestingly, briefly ramping up shear to 20 dyn/cm^2^ over 2 minutes redirected MSC lineage towards osteogenesis [7, 25]. This was thought to trigger substantial Ca^2+^ entry through polycystin-2 mechanosensitive channels. Downstream, elevated Ca^2+^ activated the CaMK-NFAT pathway to augment RUNX2 and suppress SOX9 expression. In osteoblasts, low FSS of 5 dyn/cm^2^ enhanced proliferation and differentiation through Integrin/FAK/ERK signalling [26]. This shear optimized bone nodule formation. Much higher FSS exceeding 20 dyn/cm^2^ inhibited osteoblast mineralization potentially by disrupting integrin-mediated mechanotransduction or inducing apoptosis [9]. Together, these studies showed the imperative of tightly regulating FSS magnitude during bioreactor culture, as even subtle differences in shear could determine cell fate in musculoskeletal tissues like cartilage and bone. Furthermore, excessive FSS (> 10-fold upregulation) can lead to increased H3K4me3, targeting ZBTB20 and activating Wnt signaling, contributing to cartilage degeneration [10]. This highlights the epigenetic influence of FSS on cartilage. While biomaterials like collagen microcarriers or nanocomposite xerogels can enhance MSC viability and migration, and even osteogenic differentiation when combined with FSS [11], the interplay between FSS and biomaterials in directing MSC chondrogenic differentiation requires further investigation. In this study, we confirmed that low FSS protects chondrocytes by inhibiting IL-1β-induced chondrocyte apoptosis.

Our findings demonstrate that low FSS protects chondrocytes from IL-1β-induced apoptosis via the miR-143-3p/ERK5/KLF4 axis. This mechanism adds to our understanding of the protective effects of mechanical stimulation on cartilage health. This observation is consistent with the growing body of literature highlighting the crucial role of non-coding RNAs, such as microRNAs (miRNAs) and small interfering RNAs (siRNAs), in the pathogenesis and treatment of various musculoskeletal diseases. For instance, in tendon injuries, miRNAs have been shown to regulate cytokine expression and influence the behavior of cells involved in extracellular matrix composition [27]. Similarly, in rheumatoid arthritis (RA), siRNAs have proven useful in identifying therapeutic targets and studying inflammatory processes [28], and in the context of tendon healing, siRNAs have been implicated in regulating genes encoding for structural molecules of tendon fibers [29]. The involvement of miRNAs in osteoarthritis (OA) has also been extensively documented, with complex interactions between miRNAs and their target genes influencing gene regulation and homeostatic pathways [30]. While our study focuses on the protective role of low FSS and miR-143-3p in chondrocytes, the broader implication is that manipulating non-coding RNA expression, similar to the effects of low FSS observed here, might offer novel therapeutic strategies for cartilage-related diseases. This aligns with the potential of siRNAs in osteoporosis research, where they are being explored as tools for identifying therapeutic targets for novel drug therapies [31]. Further research is needed to explore the translational potential of these findings and to investigate whether targeting specific non-coding RNAs could be used to enhance cartilage repair and regeneration.

MiR-143-3p has diverse regulatory roles in different tissues and conditions [12, 13, 32, 33]. In the vasculature, miR-143-3p is involved in vessel remodeling upon injury [34]. Elevated fluid shear stress stimulates endothelial cells to secrete TGF-β, activating SMAD expression in smooth muscle cells and upregulating miR-143-3p. This regulates extracellular matrix remodeling by downregulating collagen V synthesis and promoting angiogenesis [34]. In leukemic cells, decreased miR-143-3p expression promotes proliferation. miR-143-3p inhibits viability and growth of leukemic cells by downregulating Histone acetyltransferase 6 A [35]. Polycystic ovary syndrome follicular dysplasia is also associated with miR-143-3p, as FF-derived miR-143-3p suppresses glycolysis in GCs, affecting survival, apoptosis and impaired follicle development [32]. In joint tissues, miR-143-3p seems to play a different role. Compared to healthy tissues, miR-143-3p decreases in osteoarthritic lesions with no significant reduction following IL-1β treatment [16]. Our study showed similar effects. In summary, miR-143-3p exerts diverse regulatory functions in different cell types and tissues by modulating various targets and pathways. It plays important roles in disease pathogenesis through complex mechanisms warranting further elucidation.

Mitogen-activated protein kinases (MAPKs), such as ERK5, play important roles in transmitting extracellular signals and regulating cellular processes [36]. ERK5 is involved in key physiological functions and diseases like cancer [37]. Recent research found the ERK5 pathway serves as a major survival and proliferation escape route for tumor cells under drug stress [18]. Dysregulated ERK5 also promotes cardiovascular diseases such as atherosclerosis. Our study showed low fluid shear stress upregulates ERK5 expression, suppressing IL-1β-induced chondrocyte apoptosis. Krüppel-like factor 4 (KLF4) regulates processes including cell growth, proliferation and differentiation. Since identified as one of four factors inducing stem cell differentiation in 2006, KLF4 attracted significant research interest [38]. KLF4 protects cartilage by enhancing chondrogenesis and suppressing hypertrophy. Long non-coding RNA MEG3 prevents IL-1β-induced chondrocyte inflammation by controlling the miR-9-5p/KLF4 axis [39]. Our research demonstrated low fluid shear stress upregulates the protective ERK5/KLF4 axis against IL-1β-induced chondrocyte apoptosis.

Several limitations of this study warrant consideration. Firstly, while the simplified culture conditions ensured the controllability and reliability of the study, they may not fully recapitulate the intricate interplay of various cell types, growth factors, and mechanical forces present within the in vivo environment of articular cartilage. Further in vivo studies are necessary to validate the protective effects of low FSS and the role of the miR-143-3p/ERK5/KLF4 pathway in mitigating IL-1β-induced cartilage damage in a more physiological context. Secondly, while we focused on the miR-143-3p/ERK5/KLF4 pathway, other signaling cascades may also contribute to the chondroprotective effects of low FSS and the response to IL-1β. Our study does not exclude the involvement of other miRNAs or signaling pathways that could be interacting with or modulating the observed effects. Further investigation is needed to fully elucidate the complex network of molecular events involved.

In summary, we showed that miR-143-3p was significantly downregulated in SW1353 cells in response to low FSS. Additionally, miR-143-3p downregulation prevented IL-1β-induced apoptosis in chondrocytes. Furthermore, we identified ERK5 as a direct target of miR-143-3p. These findings may offer novel insights for further investigation into mechanically-induced chondrocyte culture.

## Data Availability

No datasets were generated or analysed during the current study.
